# Sensing Biomechanical Alterations in Red Blood Cells of Type 1 Diabetes Patients: Potential Markers for Microvascular Complications

**DOI:** 10.3390/bios14120587

**Published:** 2024-12-02

**Authors:** Riccardo Di Santo, Benedetta Niccolini, Alessandro Rizzi, Laura Bertini, Denise Pires Marafon, Maria Vaccaro, Federica Cristallo, Enrico Rosa, Linda Tartaglione, Laura Leo, Marco De Spirito, Gabriele Ciasca, Dario Pitocco

**Affiliations:** 1 Department of Life Science, Health, and Health Professions, Link Campus University, 00165 Rome, Italy; 2 Dipartimento di Neuroscienze, Sezione di Fisica, Università Cattolica del Sacro Cuore, 00168 Rome, Italy; 3 UOSA Diabetologia, Fondazione IRCCS, University Agostino Gemelli, 00168 Rome, Italy; alessandro.rizzi@unicatt.it (A.R.); dario.pitocco@policlinicogemelli.it (D.P.); 4 Section of Hygiene, Department of Life Sciences and Public Health, Università Cattolica del Sacro Cuore, 00168 Rome, Italy; 5 Fondazione Policlinico Universitario “A. Gemelli”, Istituto di Ricovero e Cura a Carattere Scientifico (IRCCS), 00168 Rome, Italy; 6 Department of Theoretical and Applied Sciences, eCampus University, 22060 Novedrate, Italy

**Keywords:** Atomic Force Microscopy, biomechanics, red blood cells, diabetes, microvascular complications, biomarkers, blood biochemistry

## Abstract

In physiological conditions, red blood cells (RBCs) demonstrate remarkable deformability, allowing them to undergo considerable deformation when passing through the microcirculation. However, this deformability is compromised in Type 1 diabetes mellitus (T1DM) and related pathological conditions. This study aims to investigate the biomechanical properties of RBCs in T1DM patients, focusing on identifying significant mechanical alterations associated with microvascular complications (MCs). We conducted a case-control study involving 38 T1DM subjects recruited from the Diabetes Care Unit at Fondazione Policlinico Gemelli Hospital, comprising 22 without MCs (control group) and 16 with MCs (pathological group). Atomic Force Microscopy was employed to assess RBC biomechanical properties in a liquid environment. We observed significant RBC stiffening in individuals with MCs, particularly during large indentations that mimic microcirculatory deformations. Univariate analysis unveiled significant differences in RBC stiffness (median difference 0.0006 N/m, *p* = 0.012) and RBC counts (median difference −0.39 × 10^12^/L, *p* = 0.009) between the MC and control groups. Bivariate logistic regression further demonstrated that combining these parameters could effectively discriminate between MC and non-MC conditions, achieving an AUC of 0.82 (95% CI: 0.67–0.97). These findings reveal the potential of RBC biomechanical properties as diagnostic and monitoring tools in diabetes research. Exploring RBC mechanical alterations may lead to the development of novel biomarkers, which, in combination with clinical markers, could facilitate the early diagnosis of diabetes-related complications.

## 1. Introduction

Diabetes mellitus (DM) is a prevalent chronic disorder affecting approximately 537 million individuals worldwide in 2021, rising to more than 780 million in 2045 [1]. Hyperglycemia is the primary hallmark of this metabolic condition [2] and is closely associated with substantial morbidity and mortality rates [3]. Among the complications of diabetes, microvascular complications (MCs) have a significant impact, leading to adverse effects on renal, ocular, and nervous functions [4,5,6,7]. Diabetic retinopathy (DR) and neuropathy (DN) are major microvascular complications observed in individuals with diabetes. DR is characterized by manifestations such as ocular micro-aneurysms, hemorrhages, and neovascularization [8], while DN is associated with distal sensory loss, trophic changes in the feet, and autonomic disturbances [9,10]. A previous meta-analysis demonstrated the prevalence of all types of retinopathy is 35.4%, and that of the proliferative retinopathy is 7.5% while DN progressively affects at least 50% of patients with both types of diabetes over time [9,11].

Given the substantial burden of MCs, there is an increasing focus on their prevention from a public health perspective [12]. Accordingly, ongoing research is dedicated to identifying key indicators for these complications in diabetes. Clinical parameters for predicting MC onset include glycated hemoglobin (HbA1c) levels, glycemic variability profiles from continuous glucose monitoring devices, blood pressure, renal function, diabetes duration, heightened oxidative stress, and concurrent medical conditions such as hyperlipidemia [12,13,14,15,16,17,18,19,20,21,22,23]. 

In this context, assessing changes in blood composition appears a major source of these markers, with red blood cells (RBCs) serving as key sensors for these changes [17,18,22,24,25,26,27,28,29,30,31,32].

Under physiological conditions, RBCs are characterized by unique mechanical properties that enable them to undergo substantial deformations while traversing the microcirculation, with vessels and capillaries that are often smaller than their size [33,34,35,36]. On the microscale, this extraordinary deformability results from multiple factors, including the high surface-to-volume ratio of RBCs and the absence of a nucleus, which is the stiffer part of a cell [33]. On the nanoscale, RBC elastic properties are derived from a distinctive spectrin network constituting the cellular skeleton, tethered to the lipid membrane bilayer by integral and peripheral proteins [37]. This intricate molecular network, dynamically changing in response to shear stress, plays a crucial role in preserving cell integrity even after repeated deformations [33,34,36]. 

In diabetes, persistent hyperglycemia raises intracellular glucose levels through passive diffusion facilitated by insulin-independent glucose transporters [38,39]. This leads to detrimental biochemical changes in RBCs, including accelerated glucose metabolism, which modifies the production of NADPH, glutathione reductase, and GSH, alongside alterations in ATP consumption [40,41]. Additionally, the formation of Advanced Glycation End Products (AGEs) involves hemoglobin, cytoplasmic proteins, and components of the plasmalemma [42]. Dysregulated eryptosis—the programmed death of RBCs—is also observed, associated with altered Ca^2+^ influx, osmotic shock, oxidative stress, and various kinase activities [43,44].

Moreover, hyperglycemia raises reactive oxygen species (ROS) levels, significantly impacting RBCs, which are highly susceptible to oxidative stress [45]. Due to both endogenous and exogenous factors, ROS levels within the cells become so elevated that the internal antioxidant systems are no longer effective [25]. In this context, Buys et al. observed significant glycosylation of cytoskeletal proteins and oxidative damage to spectrin molecules in RBCs from diabetes mellitus (DM) patients [42]. Furthermore, the glycosylation of membranous proteins irreversibly cross-links cytoskeletal proteins, affecting RBC elasticity and functionality [46]. 

These biochemical alterations deeply affect RBC biomechanics, which retains a long-term memory of such changes throughout the entire erythrocyte lifespan. This suggests that analyzing RBC biomechanics could provide potential circulating biomarkers for this pathology [34,47,48,49,50]. 

In this context, Atomic Force Microscopy (AFM) has proven to be an effective tool for studying cell mechanics at the nanoscale level [35,51,52,53,54,55,56,57,58,59,60]. Regarding RBCs in DM, previous AFM studies have demonstrated an increase in RBC rigidity [42,47,48,50,51,61,62,63,64] and an alteration in RBC dissipative properties in DM patients compared to healthy subjects [34,65,66]. A comprehensive review on this topic has been presented by Loyola-Leyva et al. [55]. 

These mechanical alterations, in turn, can disrupt blood hemorheology, both in large vessels and in the microvasculature, leading to inadequate tissue perfusion, ischemia, and hypoxia, and significantly impairing blood circulation and oxygen transport [67,68,69]. 

In the macrocirculation, the deformability and adhesion properties of RBCs significantly contribute to overall blood viscosity. In healthy conditions and at high shear rates, RBCs exhibit a specific motion known as tank-treading, preventing their accumulation in peripheral blood vessels and reducing friction with the epithelial wall [36,70]. Mechanical deformability is crucial in this process, as healthy RBCs need to elongate and decrease in thickness, a process hindered in diabetic conditions due to RBC stiffening. At low shear forces, RBCs undergo reversible aggregation, forming multicellular stacks known as *rouleaux*, impacting blood viscosity. In diabetes, this balance is disturbed, leading to increased aggregation, disrupting blood flow, and promoting RBC adhesion to the endothelial wall [36,60]. 

The impact of impaired mechanical properties on microvascular hemodynamics still remains a topic of discussion in the literature. Recent computational simulations of in-silico 3D microvascular networks, designed to resemble physiologically realistic microcirculation, suggest that decreased RBC deformability leads to detrimental blood flow alterations, primarily occurring at vascular bifurcations. Less deformable cells tend to spend less time at the majority of bifurcations, increasing the fraction of RBCs entering higher flow branches and altering wall shear stress within vessels and near vascular bifurcations. These heterogeneous and focal changes in hemodynamics may contribute to morphological abnormalities observed in capillary vessel networks, particularly in diseases like DR and DN [71].

The primary aim of this study is to assess, in support of the previously mentioned computational models, the existence of subtle alterations in the mechanical response of erythrocytes in DM patients diagnosed with MCs. To achieve this goal, we employ a case-control study design, recruiting 22 T1DM subjects without evidence of MCs (control group) and 16 subjects diagnosed with MCs, specifically DR, DN, or both. The erythrocyte mechanical properties are mapped using a single-cell approach, employing AFM in force spectroscopy mode. The results of this study provide additional evidence of the occurrence of these mechanical alterations, paving the way for the search for novel potential circulating mechanical markers of diabetic microvascular complications.

## 2. Materials and Methods

### 2.1. Patient Recruitment and Study Population

For this case-control study, 38 participants, 9 females (23.7%), 29 males (76.3%), with a median age of 44 years (range: 20–75 years) were enrolled, including 22 T1DM patients without MCs and 16 patients with a confirmed diagnosis of MCs (3 with DR, 8 with DN, and 5 with both DN and DR). The first group served as the control, while the second was designated as the pathological group. MCs were diagnosed following the 2020 guidelines set by the American Diabetes Association (ADA) [72]. To evaluate the presence of nephropathy, spot urinary albumin-to-creatinine ratio and estimated glomerular filtration rate [eGFR] were assessed while dilated retinal examinations were performed to assess the presence of diabetic retinopathy. The presence of distal symmetric polyneuropathy (DPSN) was assessed through the evaluation of vibration sensation using a 128-Hz tuning fork and pinprick sensation and electrophysiological tests to evaluate nerve conduction (amplitude, conduction velocity). The study was conducted after obtaining approval from the local ethical committee (protocol number: ID5860). Participants for this research were chosen from the pool of patients attending the Diabetes Care Unit at the Fondazione Policlinico Gemelli (FPG) Hospital, IRCSS (Rome, Italy). All clinical investigations adhered to the principles outlined in the Declaration of Helsinki, with participants providing informed consent for their participation in the study. Patients under 18 years old, pregnant women, and those participating in clinical trials involving drug usage were excluded from the study. Included patients had a Body Mass Index (BMI) ranging from 18.5 to 30 kg/m^2^, had systolic and diastolic blood pressure below 140 and 90 mmHg, and displayed adherence to therapeutic treatment. The clinical and demographic characteristics of the patients, along with the biochemical and mechanical parameters are summarized in Table 1. Despite a higher prevalence of men observed in both groups compared to women, no statistically significant differences were observed for sex (*p* = 0.40) and age (*p* > 0.90) between the two groups. 

### 2.2. Sample Preparation and Atomic Force Microscopy Measurements

Human whole blood was obtained via venipuncture at the Fondazione Policlinico Gemelli Hospital in Rome, collected in heparinized tubes, and centrifuged at 500× *g* for 10 min to separate the blood from the serum. Samples were used promptly after collection to assess both biochemical markers, following the standard clinical pathway for patients, and mechanical properties.

Specifically, to evaluate RBC mechanical properties using AFM, erythrocytes were suspended in 10 mM phosphate-buffered saline (PBS, 150 mM NaCl, 27 mM KCl, pH = 7.4, from Sigma, Tokyo, Japan) and deposited onto a poly-L-lysine-coated Petri dish, as described in previous studies [34,53,58]. After an hour of incubation, the Petri dish was gently washed with PBS to remove unattached RBCs.

Atomic force microscopy experiments were conducted in a liquid environment using a JPK Nanowizard II atomic force microscope (JPK Instruments, Berlin, Germany) coupled with an optical microscope (Axio Observer, Carl Zeiss, Milan, Italy). MikroMash silicon cantilevers, with a spring constant of approximately 0.05 N m^−1^ and a tip radius of about 10 nm (CSC38, MikroMash), were utilized. The cantilever spring constant was determined for each measurement through thermal calibration. Notably, the height measured from the contact point at the maximum height on red blood cells shows no statistically significant difference between controls (Mean = 2.64 µm, 95% CI 2.00–3.27) and patients with microcomplications (Mean = 2.53 µm, 95% CI 1.91–3.14).

**Figure 1:**
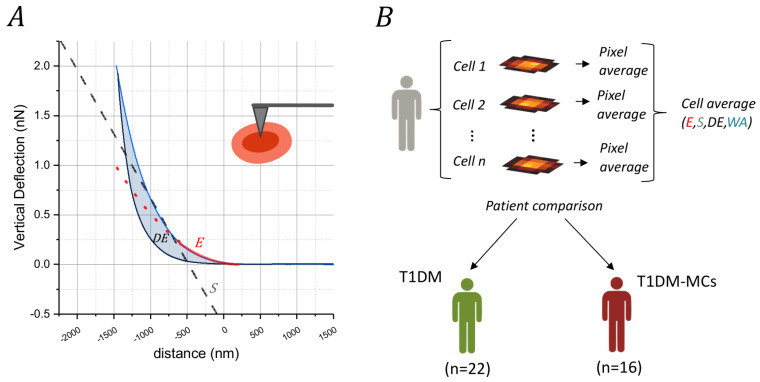
Representative force–distance (FD) cycles (blue: approach phase; black: retraction phase) acquired on a red blood cell obtained from a pathological subject (**A**). The cyan shaded area represents dissipated energy and hysteresis, while the red continuous line represents the best fit of the curve using the Sneddon model. The dashed black oblique line represents the slope (S) measured in N/m of the FD approach curve, which also provides information on the cell stiffness. A schematic view of the AFM tip indenting the cell is shown in the upper right corner. Schematic representation of the design of the experiment (**B**).

Force–distance (FD) cycles (Figure 1A) were recorded using an indentation force of 2 nN at an indentation rate of 5 μm s^−1^. Accordingly, the total indentation depth of the red blood cell varies depending on its stiffness and the position at which the curve was acquired. At least 10 randomly selected cells were measured for each patient, localized based on optical microscopy images. We acquired a map of 64 FD curves for each cell (Figure 1B), with each map covering the whole RBC surface and a portion of the unoccupied Petri surface. The latter region was eliminated before data analysis. FD approach curves were analyzed using the Sneddon model (Figure 1A, red continuous line) [73]:

(1) $$F\left(\delta \right)=\frac{2E\;tan(\alpha )}{\pi \;(1-\nu^{2})}\delta^{2}$$

where E accounts for the apparent Young’s modulus, υ for the Poisson ratio, and δ for the indentation depth. The Poisson ratio was set at 0.5 to account for material incompressibility. For each force–distance curve, Young’s modulus was obtained by fitting the initial parabolic region of the curve in the negative portion. Specifically, Equation (1) was fitted to the experimental data in the 0–250 pN range, corresponding to an indentation depth of several hundred nanometers. A typical example is shown in Figure 1A, where an indentation depth of approximately 600 nm is observed. 

Aside from the apparent Young’s modulus E, other mechanical quantities were computed from the analysis of the force–distance curves, including hysteresis (H), energy dissipated during indentation (DE, Figure 1A blue shaded area), work adhesion (WA, Figure S1 dark green shaded area), and the slope of the FD curve during indentation (S, Figure 1, oblique dashed line). 

Energy dissipated during indentation (DE) is computed as the difference between the area under the approach curve F_A_(δ) and the retract curve F_R_(δ):

(2) $$ED=\int_{0}^{\delta_{max}}F_{A}\left(\delta \right)d\delta -\int_{0}^{\delta_{max}}F_{R}\left(\delta \right)d\delta =A_{E}-A_{R}$$

The hysteresis (H), which represents the perceptual energy dissipated during indentation, was computed by normalizing Equation (2) as follows [74,75,76,77]:

(3) $$H=\frac{\int_{0}^{\delta_{max}}F_{A}\left(\delta \right)d\delta -\int_{0}^{\delta_{max}}F_{R}\left(\delta \right)d\delta }{\int_{0}^{\delta_{max}}F_{A}\left(\delta \right)d\delta }=\frac{A_{E}-A_{R}}{A_{E}}$$

Work of adhesion (WA) is measured as the area under the FD cycle during the retraction phase of the indenter. It represents the total energy required to separate the AFM probe from the sampled surface after indentation, thus providing insights into the adhesive interaction between the AFM probe and the sample during the experiment. As discussed in more detail in the experimental results and discussions, the vast majority of the curves examined are characterized by negligible work of adhesion. Only sporadically (see the curve in Figure S1) do we obtain curves with measurable work of adhesion, almost exclusively in patients with microcomplications—a phenomenon we hypothesize may be due to the increased fragility of these cells.

In addition, we measured the slope (S) of the linear portion of the force–distance curve recorded during the sample indentation. S, measured in N/m, represents the force required to achieve a given deformation of the sample, thereby providing information about its stiffness. According to this definition and its physical units, S will be referred to as “AFM stiffness” in the following. S was obtained by fitting the portion of the curve within the indentation force range of 250–750 pN, which extends beyond the previously defined parabolic indentation region and shows an approximately linear behavior. The selection of this specific region for analysis was based on its potential diagnostic utility, as further commented in the discussion section, rather than the derivation of a precise material property of the red blood cell. We acknowledge that this region of the curve significantly exceeds the indentation values at which the contribution of the substrate to the mechanical response of the cell can be neglected. Thus, a more rigorous analysis of the mechanical parameters would require more complex models, such as those described in the literature [78,79,80,81]. However, from the perspective of identifying a potential quantitative diagnostic marker for pathology, the influence of substrate contribution in the mechanical response is not a significant issue, provided that this contribution is consistent across all measurements and involves a substrate with identical mechanical properties, as in our study.

Similar to other diagnostic techniques widely used in medicine, such as histological evaluations, even though the method may introduce significant artifacts due to experimental conditions and produce quantitative determinations that differ from physiological conditions, it remains valid for comparing pathological and physiological states obtained under identical conditions. In this context, a simple linear model, if diagnostically useful, is more effective than complex models for accurately determining cell material parameters, as it is more easily applicable in clinical practice in an automated and reproducible manner.

The parameters H, DE, A, and S were calculated from the FD cycles using custom-made software programmed in-house, following methodologies described in prior publications [74,75,77,82,83].

### 2.3. Data Visualization and Statistical Analysis

Discrete variables are presented in terms of absolute frequency and/or percentages. Continuous variables were tested for normality using the Shapiro–Wilk test and through visual inspection of the QQ plot. Several variables, particularly mechanical parameters, exhibited significant deviations from normality. Therefore, continuous variables were represented in terms of median and interquartile range (Q1, Q3). Tabular data were reported using the R package “gtsummary” [84].

For the comparison between the two recruited groups, the Chi-square test or Fisher’s exact test was used for categorical variables, and the Wilcoxon–Mann–Whitney U test was used for continuous variables. Statistical significance was set at *p* < 0.05. 

The potential diagnostic applicability of the investigated mechanical, biochemical, and clinical parameters, both individually and in combination, was assessed through Receiver Operating Characteristic (ROC) curve analysis. Subsequently, the corresponding area under the curve (AUC) values were computed. The AUC values, along with their confidence intervals, were calculated using the De-Long method and then compared with the value of 0.5, which indicates a random classifier. The ROC-AUC analysis was conducted using the “*pROC*” package in R [85]. 

A multivariate logistic regression was performed to assess the potential confounding effect of the disease duration on the association between the occurrence of MCs and the levels of selected mechanical markers. Due to the limited sample size (38 patients divided into two groups), no more than two variables were included at a time in the multivariate analyses. Additionally, to identify a potential combined biomarker for MC diagnosis, a stepwise logistic regression was applied to select the most informative subset of markers based on the *Akaike information criteria* (AIC), according to a previous manuscript [86,87]. The stepwise procedure was conducted starting from a full model incorporating all variables showing a *p*-value < 0.25 in the univariate analysis. Given the limited sample size, we assessed the accuracy of multivariate logistic models in predicting group membership through leave-one-out cross-validation (LOOCV), utilizing the “*cv.glm*” function in R. Comparison between different ROC curves was conducted according to [88].

Correlations among biochemical, clinical, and mechanical markers were assessed using Spearman’s correlation coefficients. Only statistically significant correlations, identified through a power analysis, were reported. The significance level was set to *p* < 0.05. Significant correlation coefficients were organized in a correlation matrix using the R package “Corrplot” [89]. 

The shape of the distribution of selected parameters, specifically Young’s modulus E, was examined by generating a Cullen and Frey plot, a statistical chart where the experimental distribution kurtosis ($k_{r}$) is plotted against the experimental squared skewness ($s_{k}^{2}$). This analysis was conducted using a slightly modified version of the “*descdist*” function within the R “*fitdistr*” package [53,90]. This function incorporates a nonparametric procedure, which calculates skewness and kurtosis values from a set of bootstrapped samples generated by resampling the original distribution with replacement. 

Data visualization and statistical analyses were performed with the software packages R (version 4.2.1), OriginPro 2022, and Stata 18.0. 

## 3. Results

### 3.1. Analysis of Force–Distance Curves for Determining Mechanical Parameters

In Figure 1A, we show a representative FD cycle measured on an RBC obtained from a patient with MCs. A qualitative analysis of this figure reveals the presence of hysteresis H between the approach and the retract curve (shaded cyan area, Equation (3)), which, in line with previous studies, demonstrates the significant role of dissipative forces in the biomechanical response of RBCs [34,53,65]. The selected curve displays a negligible contribution of the work of adhesion (WA), consistent with the majority of measured curves. A force–distance curve with a non-negligible WA contribution is presented in Figure S1 (dark green shaded area). Interestingly, curves with characteristics similar to those in Figure S1 are observed sporadically and almost exclusively in pathological subjects, as discussed in Section 4.

Young’s modulus, providing information on the cell rigidity, was retrieved by fitting the FD curves with the Sneddon model (Equation (1)), and the best regression curve was superimposed on the data (red continuous curves). According to this analysis, the selected curve displays a Young’s modulus of approximately 1500 Pa. As an additional characterization of the sample’s stiffness, we assessed the slope of the approach FD curve, obtaining a value of 0.00123 ± 0.0001 N/m in the curve region between 250 and 750 pN (oblique dashed straight line, Figure 1A). 

Unfortunately, a single FD cycle cannot be considered representative of the biomechanical behavior of a patient’s cells. Therefore, we measured 10 cells per patient, acquiring 8 × 8 pixel elasticity maps. Starting from these values, the mean of the diverse elastic parameters was computed across cells, and the value was assigned to each patient, as schematically represented in Figure 1B.

### 3.2. Investigating Mechanical Differences in Red Blood Cells and Biochemical Alterations in the Blood of Type 1 Diabetes Mellitus Patients with and Without Microvascular Complications

Table 1 presents the demographic characteristics (sex and age), clinical information (duration of the disease and presence of MCs), levels of plasma biomarkers, and mechanical parameters, namely, Young’s modulus (E), stiffness (S), the energy dissipated during indentation (DE), hysteresis (H), and the adhesion work (A). The data are reported in terms of the median (Q1, Q3), and the presence of statistically significant differences was tested using a non-parametric Wilcoxon–Mann–Whitney U test for independent samples.

**Figure 2:**
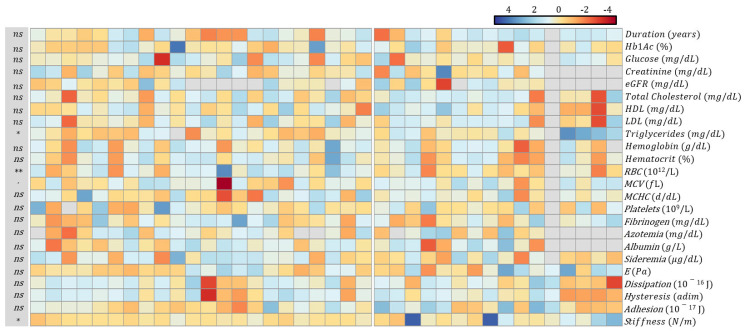
Heatmap of clinical (duration), biochemical (red blood cell indices, hematocrit, lipid, liver, glucose, and renal panel), and mechanical parameters. To enable a simultaneous visualization of all parameters, the measured values are normalized in terms of z-scores. The gray color indicates missing values, which can be considered random. Statistical significance is indicated as follows: * *p* < 0.05, ** *p* < 0.01, ∙ *p* < 0.1 and ns (not significant).

Selected continuous variables (disease duration, biochemical, and mechanical parameters) are also summarized in Figure 2 as a heatmap, with columns representing patients and rows representing markers. To enable a simultaneous visualization of all parameters, the measured values are normalized in terms of z-scores. The two groups of patients were separated by a blank gap. Missing values are represented using gray color and can be considered missing completely at random (MCAR); therefore, they should not affect the estimator values or the comparison between the two groups.

The analysis of Figure 2 and Table 1 reveals statistically significant or suggestive differences in several biochemical and mechanical parameters.

**Figure 3:**
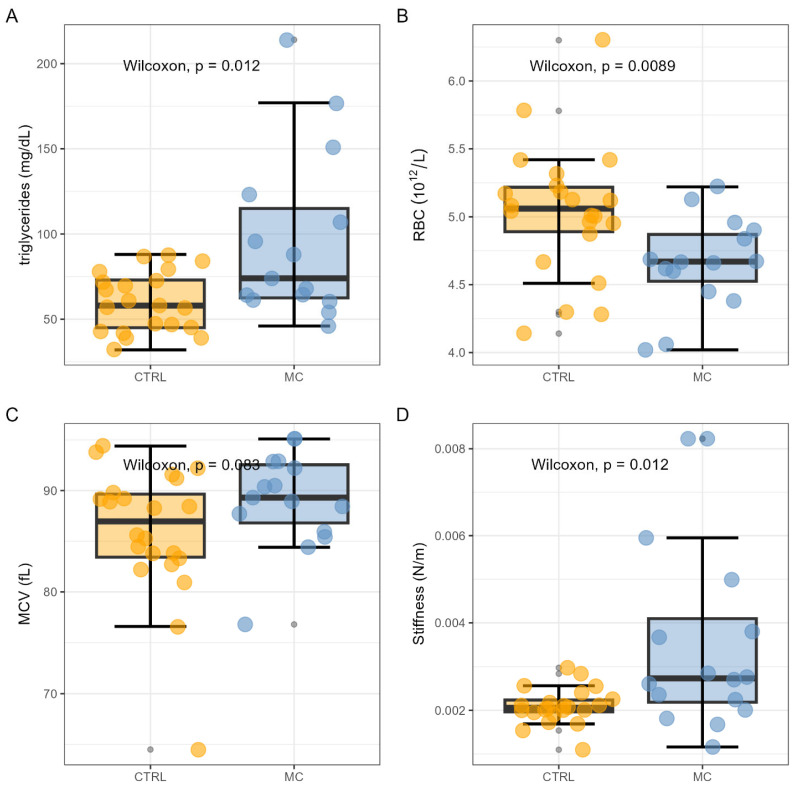
Box plot analysis of the significant variables including plasma triglyceride levels (**A**), red blood cell count (**B**), mean corpuscular volume (**C**), and AFM stiffness (**D**).

A box plot analysis of these variables is presented in Figure 3A–D. Specifically, an elevation in plasma triglyceride (TG) levels is observed in subjects with MCs compared to controls (Figure 3A, *p* = 0.012). A significant reduction in RBC count is also observed (Figure 3B, *p* = 0.0089), suggesting a potential alteration in erythropoiesis in pathological subjects. Increased MCV values, albeit not statistically significant (*p* = 0.083), are shown in Figure 3C. Particularly interesting for our purposes, a significant difference is noted in the values of the mean RBC stiffness, which is increased in subjects with complications (*p* = 0.012, Figure 3D). 

In Figure S2, we examine the behavior of four additional relevant variables: glycated hemoglobin (Figure S2A), disease duration (Figure S2B), plasma levels of LDL (Figure S2C), and the apparent Young’s modulus E (Figure S2D). While these variables did not achieve statistical significance, they warrant a more detailed discussion. Despite elevated HbA1c being considered a risk factor for MC development [12], this parameter is not increased in the pathological group (Figure S2A). As expected, a longer, albeit not significantly different, disease duration is observed in patients with complications compared to the control group (Figure S2B). Although there are no significant differences (*p* = 0.68) or even suggested differences, plasma LDL levels in the control group display a median value below the recommended 100 mg/dl threshold for primary prevention of cardiovascular events [91,92]. In contrast, the pathological group exhibits median levels of plasma LDL above this threshold—an interesting finding that deserves a more in-depth study.

**Figure 4:**
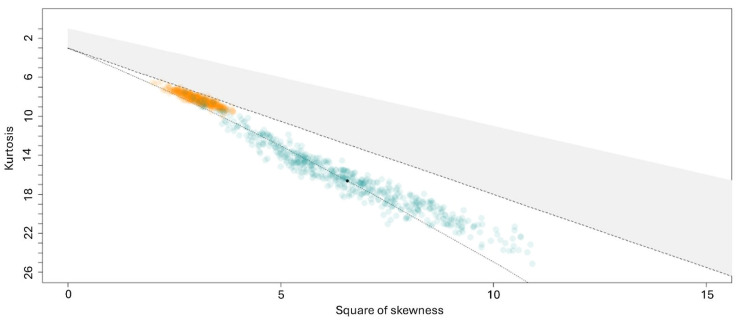
Cullen and Frey Plot showing the potential distribution shape of E values for RBCs extracted from complicated (green) and non-complicated subjects (gold). The dotted line indicates the plot region corresponding to a log-normal distribution, the dashed line corresponds to the Gamma distribution, and the shaded gray region corresponds to the family of beta distributions.

Finally, despite being elevated in pathological subjects (median difference, ΔE, of approximately 250 Pa), Young’s modulus does not demonstrate statistically significant differences between the two groups (Figure S2D). This lack of significance is intriguing, as—similar to the S parameter in Figure 3D—the E value provides information on cell rigidity. To explore this aspect further, we also assess potential differences in the distribution of Young’s moduli instead of solely comparing their median values. This approach aims to determine whether the distribution of Young’s modulus can offer a more sensitive means of detecting subtle biomechanical changes in RBC biomechanics than a measure of its central tendency. For this purpose, a comparison of the experimental E distributions was conducted by computing the Cullen and Frey plot (see Section 2.3). For this analysis, measurements from cells obtained from patients in the same group were pooled together. A full dot surrounded by a gold/cyan cloud indicates the position of the average distribution of control/complicated subjects on the chart (Figure 4). Statistical errors arising from limited sampling are addressed using the bootstrap procedure described in Section 2.3. The results are visually represented by the *ensemble* of transparent dots surrounding the corresponding experimental values. Overall, Figure 4 allows us to visualize, in a model-independent manner, the differences in distribution shapes between controls and pathological subjects by identifying subtle changes in the tails of their right-skewed E distributions. In both groups, we observe a distribution shape that is compatible with a log-normal curve, consistent with prior studies [93,94]. Most importantly, it is possible to notice that the distribution of the pathological subjects exhibits both higher skewness and higher kurtosis compared to that of the controls. This suggests larger departures from a symmetric bell-shaped distribution for the pathological group, with more extreme E values in the tail (larger skewness) and a potentially sharper peak in the distribution (larger kurtosis). We also speculate that this data representation might have potential use in clinical practice, as a means to graphically visualize the presence of pathological alterations in red blood cells when their E distribution falls in the lower part of the plot [53].

### 3.3. Definition of a Potential Mechanical Biomarker, Alone and in Combination, for the Diagnosis of Microvascular Complications in Patients with Type 1 Diabetes

In addition to assessing alterations in the biomechanical properties of RBCs associated with the presence of MCs, this work also aims to identify potential biomarkers capable of predicting the onset of such complications. Therefore, we find it useful not only to identify the presence of statistically significant differences in the investigated markers but also to assess their potential ability to discriminate between patients belonging to the two groups.

**Figure 5:**
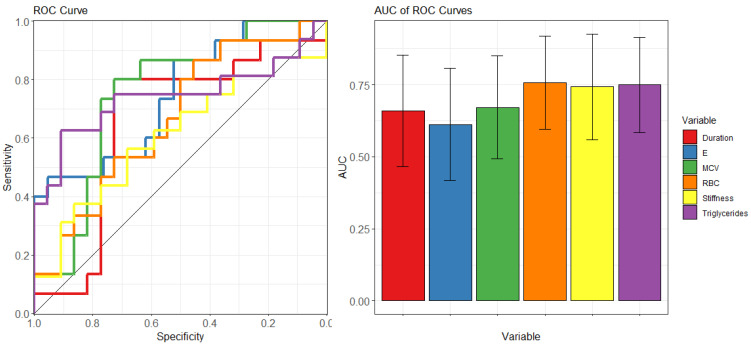
Receiver Operating Characteristic (ROC) curves illustrating the diagnostic performance of selected clinical, biochemical, and mechanical variables (**left**). The corresponding values of the area under the curve (AUC) with 95% confidence intervals are presented on the (**right**), providing a comprehensive assessment of each biomarker’s classification performance in predicting microvascular complications in patients with Type 1 diabetes mellitus.

For this purpose, we present in Figure 5 the Receiver Operating Characteristic (ROC) curves for selected variables of interest (*left*). Additionally, Figure 5 displays the corresponding values of the area under the curve (AUC) along with the corresponding 95% confidence intervals (*right*), a widely used parameter in laboratory medicine that provides a direct assessment of the biomarker’s classification performance [95].

The analysis in Figure 5 identifies stiffness, triglyceride levels, and RBC count as potential effective markers for discriminating patients with and without MCs. Specifically, these mentioned markers exhibit statistically significant AUC values characterized by a confidence interval that does not contain the value 0.5, expected for a random classifier. AUC values and their 95% confidence intervals, represented by the corresponding lower and upper limits, are summarized in Table 2.

Additionally, we conducted a multivariate logistic regression to assess the potential confounding effect of disease duration on the association between MCs and AFM stiffness, identified in Figure 5 as one of the most informative mechanical parameters. Due to the limited sample size (38 patients, with only 16 in the pathological group), we could include a maximum of two variables in the analysis. Here a *caveat* is necessary: even with only two independent variables, the interpretation of the following regressions requires extreme caution, as a sample size of at least 20 patients in the pathological group would be recommendable [96]. Therefore, the results should not be considered conclusive but rather suggestive for future studies. In Table 3, we present the outcome of a logistic regression with group membership as the dependent variable and RBC stiffness and disease duration as independent variables (model 1). The overall model is statistically significant (*p* < 0.0037, likelihood ratio test), albeit with a small percentage of explained variance (Pseudo R^2^~0.22). Despite the small sample size, a positive coefficient, approaching significance, is observed for stiffness (*p* = 0.069), while the disease duration displays a *p*-value far from significance. Overall, model 1 suggests a modest confounding effect of disease duration on stiffness, a hypothesis that needs to be verified in further studies with a larger sample size. In line with the univariate analysis in Table 1, the larger the measured stiffness in mN/m, the higher the odds of being diagnosed with MCs. Specifically, at the same disease duration, model 1 shows that for an increase of 1 mN/m in stiffness, the odds of having MCs in our group of T1DM patients is expected to be, on average, 2.97 times greater. Statistical significance is only suggested (95% CI 0.92–9.7). The corresponding unadjusted odds ratio was 3.71 with a 95% CI of 1.1 to 13.0. In Table 3, we present a second bivariate logistic model, with the presence of MCs as the dependent variable and RBC stiffness and count as independent variables (model 2). Model 2 is primarily intended to propose a potential combined circulating marker for MCs, which requires further validation in dedicated experiments with a larger sample size.

**Figure 6:**
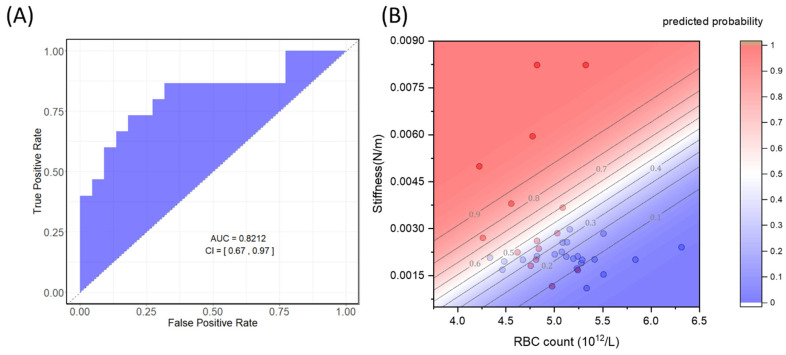
ROC and AUC analysis corresponding to the combination of the RBC stiffness and count (**A**); bivariate logistic probability function of MC occurrence according to the value of the two selected circulating biomarkers (**B**).

The combination of RBC count and stiffness was selected using step-wise logistic regression, as explained in the Section 2. In Figure 6A, we present the ROC curve associated with the combined marker introduced above, revealing an increased (albeit non-significantly; see Figure S3) area under the curve of 0.82 (95% CI: 0.74–1) compared to the case of stiffness alone (Table 2). A likelihood ratio test was conducted, comparing the full model with two parameters (stiffness and RBC count) against two nested models, each with a single variable. Statistical significance was observed in both instances when comparing the full model with stiffness alone (LR chi2(1) = 5.16, Prob > chi2 = 0.0232) and with RBC count alone (LR chi2(1) = 8.6, Prob > chi2 = 0.003).

The regression results for model 2 (Table 3) allow for estimating a bivariate probability function of being diagnosed with MC based on the joint measurements of RBC stiffness and counts, expressed by the following equation:

$$p=\frac{e^{\left(1142.357\cdot Stffness\left[\frac{N}{m}\right]-2.294652*RBC\left[\frac{10^{12}}{L}\right]\right)}}{1+e^{\left(1142.357\cdot Stffness\left[\frac{N}{m}\right]-2.294652*RBC\left[\frac{10^{12}}{L}\right]\right)}}$$

In Figure 6B, we present a contour map of the estimated bivariate probability function along with the corresponding experimental points.

Due to the small sample size, we assessed the accuracy of model 2 in predicting group membership using leave-one-out cross-validation (LOOCV), as detailed in Section 2.3, obtaining a modest accuracy of approximately 73%. 

### 3.4. Study of the Correlation Between Biochemical and Mechanical Variables

**Figure 7:**
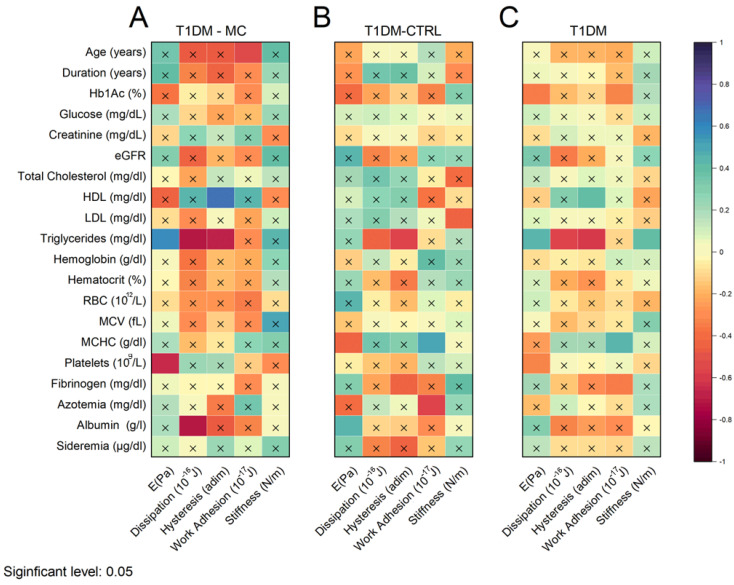
Correlation analysis between mechanical (*x*-axis) and demographic, clinical, and biochemical parameters in T1DM patients with MCs (**A**), without MCs (**B**), and in all patients recruited in the study (**C**).

In Figure 7, we present the outcomes of a correlational analysis involving all the investigated mechanical parameters (depicted along the *x*-axis) and continuous demographic, clinical factors (age, disease duration), and biochemical indicators (depicted along the *y*-axis) for T1DM patients with microvascular complications (MCs) (A), T1DM patients without MCs (B), and for all participants in the study (C). The data are represented in terms of Spearman’s correlation coefficients. The significance of these correlations was determined through a power analysis, depending on the sample size and the strength of the correlation. Non-significant coefficient values are marked with an “x”.

**Figure 8:**
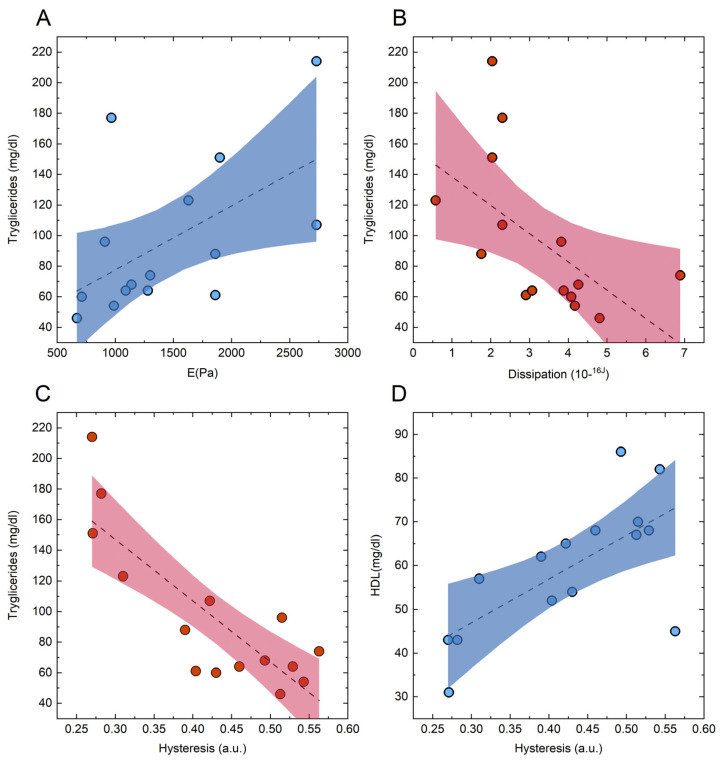
Scatter plot of selected circulating lipid markers in MC patients as a function of mechanical parameters. Triglycerides show a positive correlation with stiffness (**A**) and negative correlations with both dissipation (**B**) and hysteresis (**C**). HDL displays a positive correlation with hysteresis (**D**). The shaded areas represent the 95% confidence intervals, and the dashed lines indicate the best-fit regression lines. Data points correspond to individual MC patients.

A qualitative examination of the displayed matrices reveals some significant correlations between RBC mechanical parameters and the investigated clinical indicators. Particularly noteworthy are the correlations between plasma triglyceride levels and mechanical parameters in subjects with a diagnosis of MCs, including Young’s modulus (E), energy dissipated during indentation, and hysteresis, which also displayed a strong positive correlation with HDL levels. For the sake of completeness, the scatter plots corresponding to the mentioned correlations are reported in Figure 8, together with the best regression line along with its confidence bands. Notably, the strength and significance of correlation coefficients appear diminished in Figure 8B,C compared to Figure 8A, despite coefficients being computed from larger sample sizes. Statistically, this may seem counterintuitive, as the significance of correlations relies on the sample size. This apparently counterintuitive statistical result may indicate that T1DM patients with and without MCs exhibit a distinct correlation pattern between RBC mechanics and the lipid profile of patients. In particular, the relationship between these two sets of parameters appears enhanced in complicated subjects—an interesting result that is further discussed in Section 3 and deserves a more in-depth study. 

Additionally, Figure 8C establishes a potentially interesting link between RBC adhesion properties and HbA1c, a key indicator of glycemic control in patients. Overall, these results further support the hypothesis that RBC mechanical parameters may have diagnostic or prognostic value for T1DM patients.

## 4. Discussion

The goal of this study was to investigate RBC biomechanics in T1DM, with a specific focus on highlighting distinctions between individuals with and without microvascular complications. The correlation between RBC biomechanics and conventional biochemical markers was also considered, aiming for a better mechanistic understanding of these changes in RBC deformability.

Many prior experimental studies on RBC biomechanics predominantly focused on T2DM patients. In contrast, the present study centers specifically on individuals with T1DM, a choice aimed at ensuring a more controlled metabolic setting. In this clinical setting, pathological alterations are primarily associated with the lack of insulin production, which is compensated through standardized therapies, contributing to reduced inter-individual variability. 

The choice of this study population also has the additional advantage of helping to mitigate selection bias associated with hospital recruitment, a typical bias in the case-control study design we adopted. For diseases other than T1DM, there is often a selection bias associated with the severity of the condition, which may vary incrementally when recruitment is conducted through general practitioners, outpatient clinics, or hospital departments. T1DM stands as an exception, as patients, regardless of the severity and duration of the disease, undergo regular monitoring in the hospital outpatient setting. This facility is usually also responsible for managing acute complications, ensuring simultaneous access to both mild and severe cases within the same cohort of patients. 

Despite being a crucial parameter for monitoring DM, glycated hemoglobin measured in our sample does not show statistically significant differences between the two groups (Table 1 and Figure S2), unlike triglyceride levels. 

Our results are in agreement with the EURODIAB study and the meta-analysis by Cai et al. [97,98] which evaluate the association between triglyceride levels and increased rates of mortality.

These studies emphasize that a combination of higher triglyceride levels and other lipid-related parameters could serve as predictive markers for the onset of DN, a condition accounting for the majority of our complicated subjects.

The lack of significance in HbA1c should be further considered in relation to a potential interaction with other parameters, such as RBC count, which appears to be significantly higher in controls than in the pathological group. Several previous studies have observed an increase in this parameter in DM [20,21], likely associated with a stimulation of erythropoiesis as a compensatory mechanism to low oxygen levels [19]. Our control subjects exhibit a median RBC count greater than 5 million/mm^3^, with peaks exceeding 6 million/mm^3^, lying approximately in the normal range for this parameter or in its upper part (4.5–5.5 million cells per mm^3^ of blood [65]). In contrast, individuals within the pathological group exhibit a diminished RBC count, suggesting a potential failure of the compensatory mechanism discussed in [99]. We hypothesize that such a reduction in RBC count could be attributed to a decrease in the average lifespan of erythrocytes, a phenomenon previously observed in DM patients [100]. Interestingly, a shorter RBC life may actually reduce the time exposure to hyperglycemia for RBCs, potentially accounting for the similar HbA1c values observed in the two groups, while an increase was expected in MC patients. 

In Figure 3C, we also observed a suggested increase in MCV among T1DM patients with MCs compared to controls. This finding aligns with the existing literature, which demonstrates a positive association between increased MCV values and diabetic retinopathy (DR) [101]. A potential rationale behind this suggested increase is provided by Jiang et al. (2003), who documented a total of 42 RBC proteins differentially expressed in the RBC membranes of T2DM patients compared to controls [102]. Among these proteins, syntaxin 1C and arginase are included, which might be linked to changes in RBC volume and morphology.

We might hypothesize that this MCV-related morphological rearrangement accounts, at list partially, for the observed mechanical alterations in Figure 3D. Among other factors, the extreme RBC deformability is indeed attributed to the large surface area-to-volume ratio of these biconcave disks, evaluated by computing the mean surface area (MSA) divided by MCV [33]. Consequently, a change in MCV not balanced by a corresponding variation in MSA might induce the observed alteration in RBC deformability. Despite this potential rationale, we did not find any evidence supporting this claim in the correlational analysis presented in Figure 8. In this context, it is worth also mentioning the interesting paper by Kwon and Park, which establishes an association between borderline-high MCV and arterial stiffness [103].

Interestingly, the analysis of experimental data did not reveal differences in the main mechanical parameters commonly assessed through AFM indentation spectroscopy, such as dissipated energy, hysteresis, and Young’s modulus. However, statistically significant differences were observed in the stiffness parameter, measured from the slope of the force–distance approach curve. It is worth briefly commenting on potential reasons why only stiffness, rather than the more widely used Young’s modulus, yielded statistically significant results. Notably, Young’s modulus also exhibited poor classification abilities with an AUC value consistent with 0.5 (Figure 5). One possible explanation for this outcome is related to the specific region of the FD curve where these two parameters are assessed: Young’s modulus is determined in the region of small indentations, near the contact point, while the *S* parameter is analyzed in subsequent regions of the curve (0.25–0.75 nN), associated with more pronounced deformations of the cell membrane. This second scenario, characterized by higher deformations, likely offers a more representative model of in vivo conditions, given that red blood cells in the capillary microcirculation may experience substantial deformations. 

The selection of the previously mentioned region was driven by its potential relevance for diagnostic purposes, rather than for determining a precise material property of the red blood cell. We acknowledge that this region of the curve significantly exceeds the indentation values at which the contribution of the substrate to the mechanical response of the cell can be neglected. Thus, a rigorous analysis of the mechanical parameters would require more complex models, as described in the literature [78,79,80,81]. However, from the perspective of identifying a potential quantitative diagnostic marker for pathology, we believe the influence of substrate contribution in the mechanical response is not a significant issue, provided that this contribution is consistent across all measurements and involves a substrate with identical mechanical properties in both groups, as in our study. 

This approach, compared to more complex models, has an additional advantage when considering clinical use, as it is focused on identifying an easily measurable and reproducible metric.

As anticipated, we observe a longer, though non-significant, disease duration in patients with MCs compared to the control group (Table 1). The lack of statistical significance is likely associated with three patients in the pathological group having a disease duration of less than 15 years, identified as statistical outliers in the box plot analysis in Figure S1. This anomaly is linked to the fact that these three subjects can be classified as incident cases, having received the diagnosis of microvascular complications during the study, while the remaining subjects can be considered prevalent cases, having received the diagnosis before the study’s initiation.

In diabetes mellitus, disease duration is strongly correlated with the development of microvascular complications, naturally correlating with the patients’ age, especially in a type 1 diabetes mellitus clinical setting. Additionally, subject age has been demonstrated to induce RBC stiffening, leading us to consider the potential confounding effect of this parameter on the observed association between RBC stiffening and a diagnosis of microvascular complications. This was carried out with a bivariate logistic regression, although the results of this regression should be considered preliminary, as they are based on an extremely limited sample. Interestingly, this analysis provides a stiffness-related coefficient adjusted for disease duration that is similar to the coefficient obtained through a univariate logistic regression that considers only stiffness. This suggests a modest potential influence of disease duration on this mechanical parameter, a hypothesis that requires further validation in studies with a larger sample size.

Ultimately, we observed a significant correlation between several mechanical parameters and the blood lipid profile of patients. This pattern of correlation appears to be enhanced in subjects affected by microvascular complications. Several experimental papers highlight alterations in the lipid composition of the RBC plasma membrane in T1DM subjects with and without microvascular complications. For instance, Nayak et al. showed an increase in the membrane content of cholesterol in T2DM nephropathic patients compared to patients without complications and healthy controls [104]. Sailaja et al. [105] reported that the molecular architecture of the erythrocyte membrane lipid bilayer was altered in patients with diabetes mellitus. Similar alterations were observed by Bianchetti and Cordelli [106], who analyzed patients with different severities of the pathology and patients with and without diabetic retinopathy, respectively.

It is important to note that the correlation between these parameters does not necessarily imply a causal connection in either direction, i.e., whether lipid alterations in the membrane cause cell stiffening or vice versa. Although we cannot establish a causal link, which would require an experiment specifically designed for this purpose, we can propose a speculative biological mechanism that may explain this correlation and which requires further study for validation.

In this regard, we hypothesize that a possible mechanistic connection between these lipidic alterations and the elasticity of red blood cells might involve, as previously suggested for spur cell anemia [53], a direct effect of the lipid membrane composition on some RBC transmembrane proteins. While the cytoskeletal proteins, rather than the lipid component of the membrane, are the main contributors to the bending resistance offered by the RBC membrane, a different lipid composition could have an indirect effect on cytoskeletal stiffness. This effect could be mediated by membrane proteins, such as Band III, which not only regulate the cytoskeletal internal stress through conformational switches but are also embedded in the fluid mosaic of the membrane lipids, potentially influencing these conformational switches. A similar mechanism was hypothesized to account for the difference in RBC elasticity in T2DM patients as a consequence of hyperglycemia and dyslipidemia [42,48,49].

## 5. Conclusions

In summary, this study investigated the biomechanical properties of RBCs in T1DM patients to identify changes associated with microvascular complications. The analysis revealed significant RBC stiffening in patients with MCs, especially during extensive indentation simulating microcirculation conditions. A promising circulating MC marker, combining stiffness parameters and RBC count was also proposed, showing a substantial AUC value (0.82). The correlation between lipid profiles and RBC mechanical properties, notably stronger in the pathological group than in controls, emphasizes the relevance of RBC mechanical properties in monitoring diabetes and its complications. Although validation on a larger sample size is necessary, the overall findings highlight the potential utility of incorporating RBC biomechanical properties into laboratory medicine for the diagnosis and monitoring of diabetes and its associated complications.

## Figures and Tables

**Table 1:** Summary of demographic characteristics, clinical profiles, blood test results, and mechanical evaluations for the study participants diagnosed with type 1 diabetes, with (MC) and without microvascular complications (CTRL).

Characteristic	CTRL, N = 22 ^1^	MC, N = 16 ^1^	*p*-Value *^2^*
Gender			0.4
F	4 (18%)	5 (31%)	
M	18 (82%)	11 (69%)	
Age (years)	46 (32, 50)	43 (40, 48)	>0.9
Duration (years)	22 (10, 32)	33 (28, 36)	0.11
Hb1Ac (%)	7.2 (6.7, 7.7)	7.40 (6.9, 7.8)	0.8
HbA1c (mmol/mol)	55 (49, 61)	57 (51, 62)	0.8
Glucose (mg/dL)	134 (104, 180)	134 (106, 148)	0.8
Creatinine (mg/dL)	0.77 (0.67, 0.89)	0.72 (0.67, 0.85)	0.6
eGFR	105 (96, 118)	108 (101, 129)	0.5
Total Chol. (mg/dL)	170 (155, 189)	188 (170, 197)	0.2
HDL (mg/dL)	57 (52, 68)	62 (48, 68)	>0.9
LDL (mg/dL)	97 (88, 112)	112 (84, 116)	0.7
Triglycerides (mg/dL)	58 (45, 73)	74 (62, 115)	0.012
Hemoglobin (g/dL)	14.95 (13.95, 15.47)	14.10 (13.08, 15.25)	0.3
Hematocrit (%)	43.6 (40.9, 45.2)	42.1 (38.0, 44.4)	0.2
RBC (10^12^/L)	5.06 (4.89, 5.22)	4.67 (4.53, 4.87)	0.009
MCV (fL)	86.9 (83.4, 89.6)	89.3 (86.8, 92.6)	0.083
MCHC (g/dL)	33.95 (33.20, 35.03)	34.40 (33.70, 34.50)	0.8
Platelets (10^9^/L)	218 (203, 256)	213 (204, 274)	>0.9
Fibrinogen (mg/dl)	280 (248, 297)	276 (254, 295)	0.9
Azotemia (mg/dL)	15.0 (12.0, 17.0)	14.5 (11.2, 18.8)	0.9
Albumin (g/L)	42.00 (41.00, 44.00)	43.00 (40.00, 44.50)	0.8
Serum iron (µg/dL)	92 (64, 110)	63 (53, 80)	0.2
E (Pa)	1065 (938, 1385)	1290 (984, 1860)	0.2
Dissipation (10^−16^ J)	3.79 (3.11, 4.50)	3.45 (2.24, 4.19)	0.4
Hysteresis (adim)	0.48 (0.40, 0.52)	0.45 (0.37, 0.51)	0.5
Adhesion (10^−17^ J)	0.96 (0.73, 1.12)	1.03 (0.65, 1.67)	0.5
Stiffness (N/m)	0.0021 (0.0020, 0.0022)	0.0027 (0.0022, 0.0041)	0.012

^1^ n (%); Median (IQR) ^2^ Fisher’s exact test; Wilcoxon rank sum test; Pearson’s Chi-squared test.

**Table 2:** Area under the curve (AUC) with 95% confidence intervals (CI) for selected variables in the study.

Variable	AUC	(95% CI)
Duration (Years)	0.66	(0.47–0.85)
Triglycerides (mg/dL)	0.75	(0.58–0.91)
RBC count (1012/L)	0.76	(0.60–0.92)
MCV (fL)	0.67	(0.49–0.85)
E (Pa)	0.61	(0.42–0.81)
Stiffness (N/m)	0.74	(0.56–0.93)

**Table 3:** Coefficients for two logistic regression models predicting the log odds of receiving a diagnosis of microvascular complications in T1DM patients. While both models demonstrated overall significance at *p* < 0.005, the stiffness coefficient exhibited *p*-values approaching, though not reaching, the significance threshold.

	Model’s Coefficients	
Variable	Model 1	Model 2
Stiffness (mN/m)	1.09 (*p* = 0.069)	1.14 (*p* = 0.096)
Duration of the Disease (years)	0.027 (*p* = 0.38)	-
RBC count (10^12^/L)	-	−2.29 (*p* = 0.049)
Intercept	−3.82 (*p* = 0.011)	7.84 (*p* = 0.17)
*p* > $\chi^{2}$	0.0037	0.0004
Pseudo $R^{2}$	0.224	0.312

## Data Availability

Data will be made available by the corresponding author upon reasonable request.

## Supplementary Materials

The following supporting information can be downloaded at https://www.mdpi.com/article/10.3390/bios14120587/s1, Figure S1: Box plot analysis of HbA1c%, disease duration, LDL, and Young’s modulus in control and MC group; Figure S2: Comparison of ROC curves; Figure S3: Comparison of ROC curves for two different logistic model.
