# Supplementary material for: Sensing Biomechanical Alterations in Red Blood Cells of Type 1 Diabetes Patients: Potential Markers for Microvascular Complications
Source: Biosensors (Basel). 2024 Dec 2;14(12):587. doi: 10.3390/bios14120587 (PMC11674557; doi:10.3390/bios14120587)
Supplement: Supplementary file 1 [file biosensors-14-00587-s001.zip › biosensors-3215860-supplementary.pdf]

Supplementary Figures

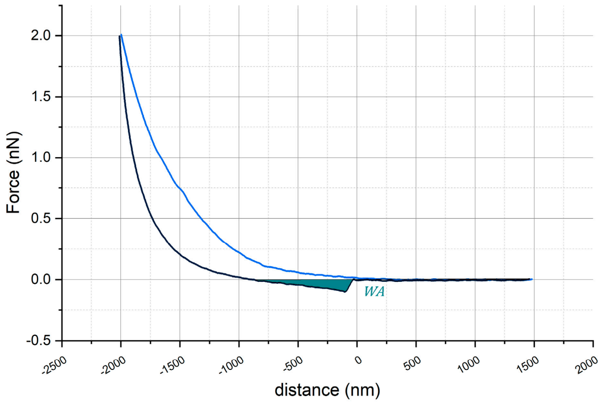

Figure S1. Force-distance curve acquired from a subject with microvascular complications, showing a non-negligible work of adhesion (WA). WA is generally negligible in the analysed red blood cells, and curves with non negligible WA have only occasionally been measured, predominantly in cells of MC subjects.

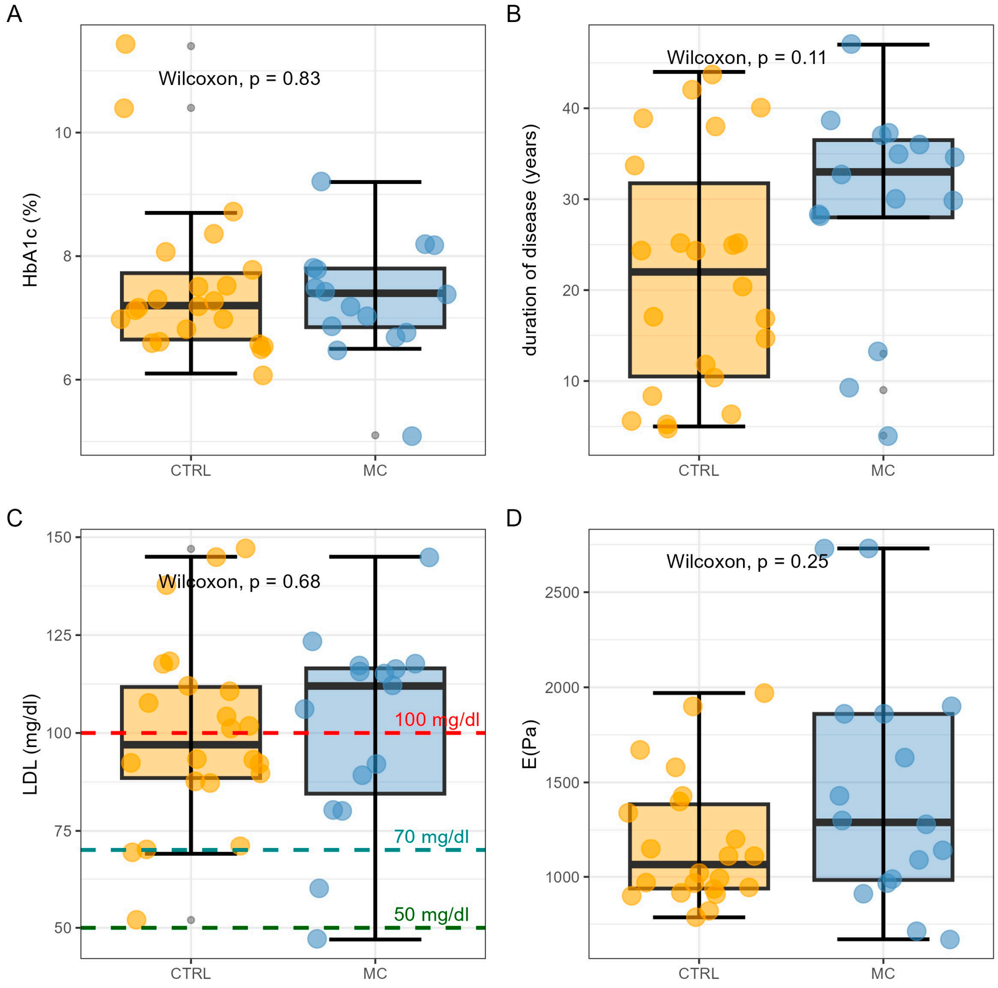

Figure S2: Box plot analysis comparing (A) glycated hemoglobin (HbA1c%) levels, (B) duration of the disease, (C) LDL levels, and (D) Young's Modulus (E) values in the two investigated groups. In 3C, measured LDL levels are reported together with suggested LDL thresholds for patients with a duration of DM of less than 5 years and in the absence of hypertension (100 mg/dL), for patients at high cardiovascular risk aged 40–75 (70 mg/dL), and for patients with three or

more cardiovascular risk factors (50 mg/dL). Interestingly, the vast majority of the recruited subjects do not meet the 70 mg/dL threshold for patients at risk of cardiovascular events. In other words, we could assert that all of these patients can be considered at risk concerning their LDL levels, irrespective of whether they belong to the control or pathological group. This suggests a potential rationale behind the lack of a significant difference in this parameter, which is widely considered a significant risk factor

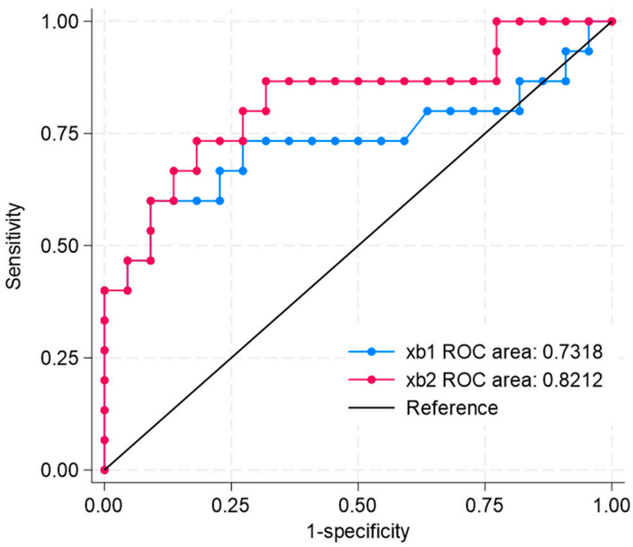

	Obs	ROC area	Std. err.	Asymptotic normal [95% conf. interval]	
Stiffness	37	0.7318	0.0984	0.53894	0.92470
Stiffness+rbc	37	0.8212	0.0762	0.67178	0.97065

H0: area(Stiffness) = area(Stiffness+rbc)
chi2(1) = 2.17 Prob>chi2 = 0.1407

Figure S3: Comparison of ROC curves for two different logistic model.
